# Modulation of Amyloid‑β Aggregation by Surface Proteins from Pathogens Associated with Alzheimer’s Disease

**DOI:** 10.1021/acschemneuro.5c00444

**Published:** 2025-08-27

**Authors:** Antonin Kunka, Hana Hribkova, Tereza Vanova, Veronika Pospisilova, Martin Havlasek, Jan Haviernik, Daniel Ruzek, Jiri Damborsky, Dasa Bohaciakova, Zbynek Prokop

**Affiliations:** † Loschmidt Laboratories, Department of Experimental Biology and RECETOX, Faculty of Science, 37748 Masaryk University, Brno 625 00, Czech Republic; ‡ International Clinical Research Center, St. Anne’s University Hospital Brno, Brno 602 00, Czech Republic; § Department of Histology and Embryology, Faculty of Medicine, Masaryk University, Brno 625 00, Czech Republic; ∥ 48357 Veterinary Research Institute, Brno 621 00, Czech Republic; ⊥ Department of Experimental Biology, Faculty of Science, Masaryk University, Brno 625 00, Czech Republic; # Institute of Parasitology, Biology Centre of the Czech Academy of Sciences, Ceske Budejovice 370 05, Czech Republic

**Keywords:** Alzheimer’s disease, amyloids, neuroinflammation, pathogen, virus, amyloid-β

## Abstract

Alzheimer’s disease (AD) is a prevalent neurodegenerative disorder. Despite substantial research efforts, our understanding of its pathogenesis remains incomplete, limiting the development of effective treatments and preventive strategies. The potential role of microbial pathogens in AD etiology has gained increasing attention. Various human microbial pathogens have been identified in the brains of AD patients, leading to the pathogen hypothesis, which posits that these microorganisms may disrupt the brain’s immune regulation and homeostasis. In this study, we examine the effects of proteins from three pathogens, *Borrelia burgdorferi*, HSV-1, and *Porphyromonas gingivalis*, on the aggregation of antimicrobial peptide amyloid-β (Aβ). Three of the four studied proteins were found to attenuate the aggregation of Aβ42 by interacting with its soluble form and inhibiting primary and secondary pathways. These in vitro findings were further supported by experiments using mature neurons derived from human pluripotent stem cells, which showed an increased accumulation of amyloid precursor protein (APP) aggregates upon infection with HSV-1 or exposure to the OspA surface protein from *B. burgdorferi*. Together, our results provide mechanistic insights into how pathogen-associated proteins modulate Aβ42 aggregation, contributing to an understanding of their potential role in AD pathogenesis.

## Graphical abstract

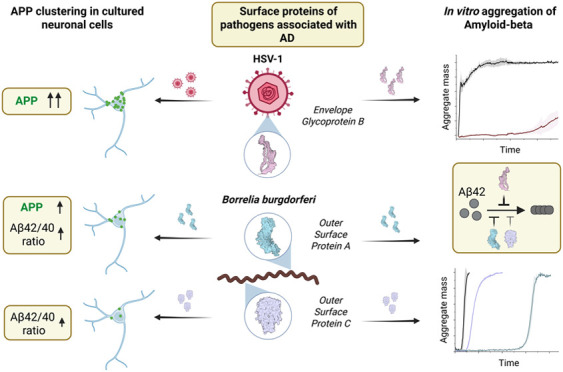

## Introduction

Alzheimer’s disease (AD) is a prevalent neurodegenerative disorder characterized by memory impairment, cognitive dysfunction, and in advanced stages, communication difficulties, disorientation, social recognition issues, and personality changes.^1^ Both sporadic and familial forms of AD exhibit these symptoms, marked by extracellular amyloid-β (Aβ42) plaques and intracellular neurofibrillary tangles of hyperphosphorylated tau protein.^2,3^ Familial AD, with an early onset, is linked to mutations in proteins involved in Aβ42 production from amyloid precursor protein (APP).^4^ The etiology of sporadic AD, accounting for over 95% of cases, is less understood, with a weak correlation between amyloid burden and cognitive impairment.^5^ Limited success in clinical trials targeting Aβ42 aggregation underscores the need to explore its function in the healthy brain and identify factors disrupting its homeostasis in sporadic AD.^6–8^ Neuroinflammation, driven by overactive microglia and astrocytes, impairs Aβ42 clearance and exacerbates neuronal damage, becoming more significant with age-related changes in the brain’s immune system.^9–13^

A potential source of neuroinflammation that has been implicated in the etiology of AD is the presence of pathogens in the brain.^1–4^ Evidence supporting the infectious cause of AD is arguably most substantial for herpes simplex virus-1 (HSV-1), which is present in the brains of a high proportion of the elderly population.^5^ The recurrent reactivation of the latent form of HSV-1 leads to cumulative damage from the neuroinflammation and, consequently, to a higher risk of AD, especially for APOE4 carriers.^6–8^ Exposure of neurons and microglia to *Borrelia* induced amyloid production and tau phosphorylation.^9^ Oral pathogen *Porphyromonas gingivalis* was identified in the AD brain tissue and stimulated Aβ42 production in animal and cell culture models.^10^ Its soluble proteases, gingipains, switch microglial activity to neuroinflammatory response, suggesting that mechanisms other than interaction with Aβ42 play a role.^11^ Other pathogens have also been implicated in AD pathology, and their links to the disease are being investigated.^12–14^ Vaccination against the herpes zoster virus was shown to have protective effects against dementia, providing further evidence of a link between infection and AD.^15,16^

The infectious hypothesis of AD is further supported by findings that Aβ42 functions as an antimicrobial peptide, showing antimicrobial activity against common pathogens.^28–30^ HSV-1 has been shown to seed Aβ42 aggregation, preventing viral entry into host cells.^13,30–32^ While definitive evidence of a causal relationship between infection and AD is lacking, these studies suggest Aβ42′s role in brain innate immunity.^33^ Prolonged or recurring infections may stimulate increased Aβ42 production, leading to its toxic accumulation, especially in individuals with impaired Aβ42 clearance, such as APOE4/4 carriers.^34,35^ Conversely, inhibiting Aβ42 production has been linked to increased infection rates, supporting its antimicrobial role.^36–38^ Understanding the interactions between Aβ42 and pathogens could therefore inform treatment and prevention strategies for AD. However, studies are needed to provide direct evidence of the interaction between proteins from AD-associated pathogens and Aβ42 aggregation.

Here, we investigate the direct effects of selected surface proteins from *B. burgdorferi* (Bb) and HSV-1, and *P. gingivalis*-soluble protease Gingipain A on Aβ42 aggregation *in vitro* and on cultured stem cell-derived neurons. We observed that HSV-1 glycoprotein B and Bb outer surface protein A increase the lag time of Aβ42 aggregation in sub- and stoichiometric ratios, respectively. The global analysis of the kinetic data further reveals that these proteins inhibit primary and secondary microscopic pathways, suggesting that they interact with monomeric Aβ42 with different potencies. Interestingly, the observed effects correlate with increased APP signaling and clustering in HSV-1-infected or OspA-treated neurons, confirming the biochemical mechanistic data observed in biological systems using infected cultured neurons.

## Results

### Selection of Proteins from Pathogens Associated with AD

In this study, we focused on pathogens that have been frequently associated with AD and selected candidate proteins based on their reported or hypothesized ability to interact with Aβ, as supported by a literature review. These include (i) Bb outer surface protein A (OspA, B7IZU3), (ii) Bb outer surface protein C (OspC, P94245), (iii) envelope glycoprotein B (glyB, P08665) from HSV-1, and (iv) gingipain R1 protease (rgpA, P28784) from *P. gingivalis* (Table 1). A detailed rationale for the selection of each protein, including its potential link to AD pathology, is provided below.

**Table 1:** Overview of Proteins from Pathogens Associated with AD Used in This Study^a^

**source organism**	*B. burgdorferi*		HSV–1	*P. gingivalis*
**protein name**	outer surface protein A	outer surface protein C	glycoprotein B	gingipain protease R1
**short name**	OspA	OspC	glyB	rgpA
**UniProt ID**	B7IZU3	P94245	P08665	P28784
**molecular weight (kDa)**	28.3	19.4	82.6	53.9
**oligomeric state**	monomer	dimer	monomer + oligomers	n.d. (monomer)
**expression**	*E. coli* (in house)	*E. coli* (in house)	mammalian (commercial)	mammalian (commercial)

a n.d. not determined experimentally, oligomeric state of the functional protein provided in the parentheses.

HSV-1 displays different types of glycoproteins on its surface that facilitate cell entry of the virus via binding to the host receptors.^17,18^ GlyB is a large (904 amino acids), multidomain protein that forms a functional trimer.^19^ It was demonstrated that Aβ interacts with glyB and inserts itself in the viral membrane in its proximity.^20^ Moreover, the protein contains a peptide fragment with sequence homology to Aβ that self-assembles into amyloid fibrils.^21^ We hypothesize that exposure of the full-length protein to Aβ in the brain can modulate its aggregation and contribute to disease pathology.^22^ To test this hypothesis, we used a commercially available construct of glyB corresponding to residues 31–774 of the full-length glyB produced recombinantly in mammalian cells by the supplier (MyBioSource). Because purified transmembrane domains of some proteins were shown to form amyloids, we used the recombinant glyB variant that lacks the *N*-terminal signal peptide and C-terminal transmembrane domains TMD and CTD, allowing its soluble expression.^23^ Using mass photometry and dynamic light scattering, we determined that the protein forms a mixture of monomers, dimers, trimers, and low-molecular-weight oligomers (ca. 500–700 kDa) under the solution conditions used in this study (Supporting Information Figure S1).

*P. gingivalis* is an oral pathogen responsible for chronic periodontitis that was found in plaques of AD patients’ brains.^10,24,25^ Specifically, the toxic proteases called gingipains, responsible for periodontal tissue destruction and evasion of host defense mechanisms, had increased load in AD brains and were found to increase production of Aβ42.^10,26^ Conversely, their inhibition by small-molecule inhibitors leads to decreased Aβ42 production.^10^ Gingipain A (RgpA) is a 991-residue-long arginine protease whose presence stimulates a strong proinflammatory response.^27^ Here, a fragment corresponding to residues 228–720 of full-length rgpA was produced recombinantly in mammalian cells by a commercial supplier (MyBioSource) and used to test its ability to modulate Aβ42 aggregation.

The outer membrane of *Borrelia* species contains several surface proteins whose composition changes in response to the spirochete environment.^28^ OspA and OspC are the major virulence factors of *Borrelia* with a strong proinflammatory response.^29–31^ They play an important role in pathogen invasion by facilitating adhesion to the host cells^32,33^ and interaction with the extracellular matrix.^34^ Both the OspA and the OspC are anchored to the outer membrane of the pathogen by lipidated cysteine in the *N*-terminus. OspA is a monomeric protein with an unusual fold comprising 21 antiparallel β strands and one α-helix.^35^ It has been demonstrated that peptide fragments derived from the central β-sheet of OspA can self-assemble into amyloid fibrils under specific solution conditions.^36,37^ As such, OspA represents a potential target for Aβ peptide during pathogen infection.^38,39^ In contrast, OspC is a dimer formed by two monomers with up and down helical bundle topology, with no evidence of amyloid-forming propensity.^40^ Both proteins were produced recombinantly in-house using protocols described in the Materials and Methods section and were experimentally verified to be predominantly monomeric (OspA) or dimeric (OspC) in solution (Supporting Information Figure S2).

### Aggregation Kinetics of Recombinant Aβ42 Is Consistent with the Mechanism Dominated by Saturating Secondary Nucleation

To minimize potential sources of irreproducibility often encountered in studies involving aggregation of Aβ42, we produced the peptide recombinantly using a well-established protocol that yields highly pure and monomeric Aβ42 ^41,42^ (Supporting Information Figures S3 and S4). Peptide aggregation was triggered by transferring the SEC-isolated monomer from ice to the assay temperature. To verify how the aggregation behavior of recombinant Aβ42 produced in our lab compares to the data described in the literature, we performed the scaling analysis of Aβ42 aggregation collected from six different data sets used in this work (Figure 1). Two representative data sets are shown in Figure 1a,b. Interestingly, the resulting double logarithmic plot was nonlinear and displayed convex shape with the scaling exponent shifting from −1.6 at low monomer concentrations (0.5–3 μM) to −0.5 at high-concentration regime (3–26 μM) (Figure 1c). The scaling coefficient of −1.6 is close to the −1.5 corresponding to secondary nucleation with a nucleus size of *n* = 2 (−(*n*+1)/2) described for Aβ42 under similar experimental conditions.^43^ The low monomer dependency of the aggregation (scaling factor −0.5) at the high-concentration regime is consistent with saturating secondary nucleation dominant mechanisms (Figure 1d,e). Further details of the kinetic analysis are provided in the Supporting Information. The fact that several independent data sets measured at different times with different batches of Aβ42 are consistent with a single model of microscopic aggregation pathways is strong evidence for the data reproducibility and provides the framework for subsequent mechanistic study of the influence of the proteins from pathogens on Aβ42 aggregation.

**Figure 1:**
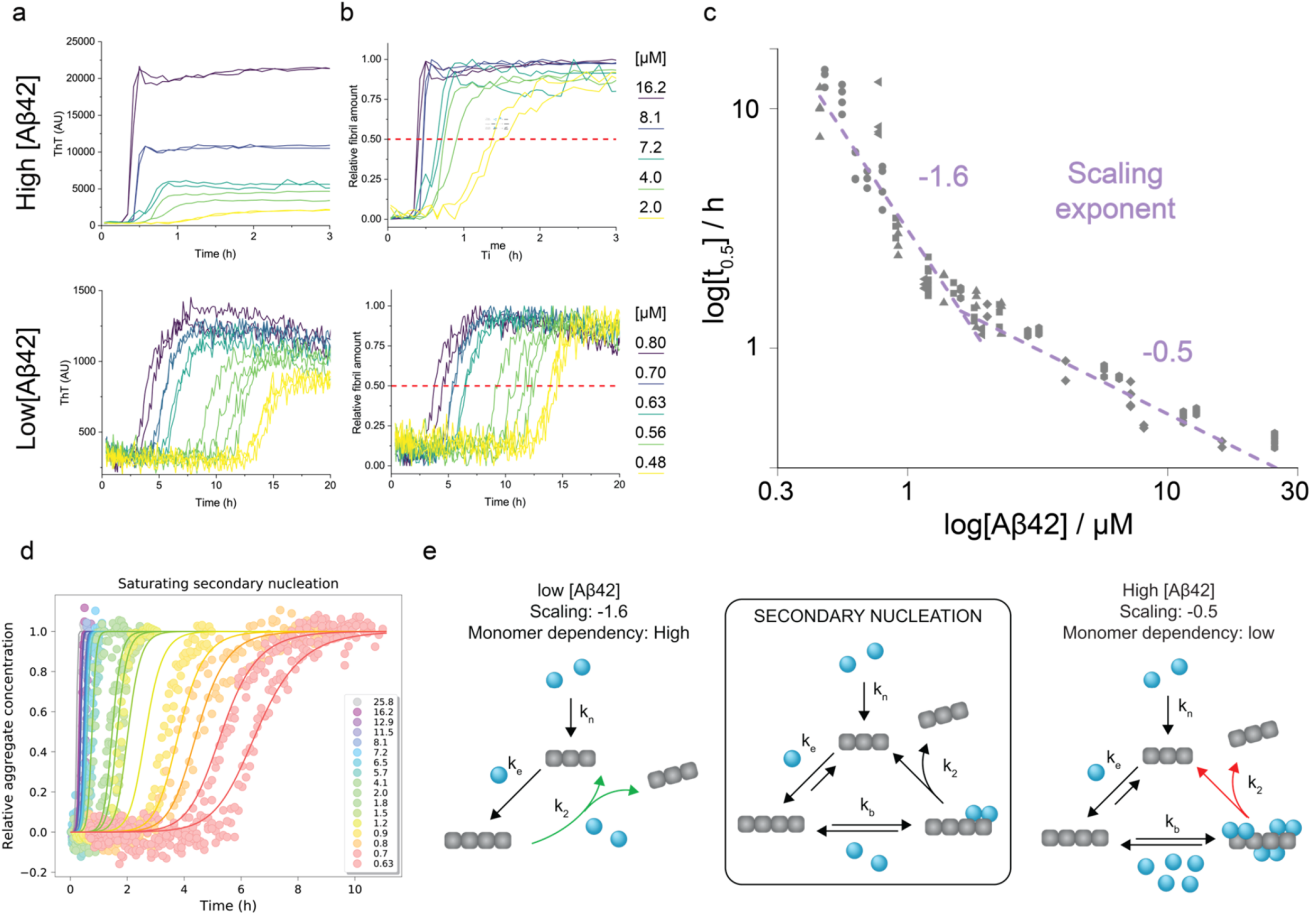
Aggregation kinetics of recombinant Aβ42 are consistent with the mechanism dominated by saturating secondary nucleation. (a) Raw ThT data at high (2–16 μM, top) and low (0.5–0.8 μM, bottom) initial Aβ42 monomer concentration. Prior to each experiment, the monomeric peptide was isolated using size exclusion chromatography and kept on ice; aggregation was then initiated by transferring the sample to an assay temperature of 37 °C. (b) Normalized ThT data. The plots in A were normalized to reflect the concentration of fibrils formed from an initially monomeric sample. Intercepts of the individual kinetic traces with the red dashed lines correspond to the half-times of aggregation. (c) Scaling of aggregation half-times with monomer concentration. Individual data points correspond to half-times extracted from six independent data sets (represented by different symbols) measured over the span of three months. The slope of the double logarithmic plot (scaling exponent) reports on the dominant microscopic pathway involved in the aggregation.^44^ The nonlinearity of the plot indicates a switch between high and low monomer dependency of Aβ42 aggregation kinetics caused by the change of the rate-limiting step of the dominant mechanism. (d) Global fit of the data sets into the multistep secondary nucleation model using AmyloFit v2.0. The saturation constant (*K*_m_) was fixed during the analysis. (e) Schematic illustration of the processes involved in the aggregation of Aβ42. The aggregation involves the formation of the primary nucleus (*k_n_* = nucleation rate) from a monomeric sample (blue spheres), which is converted into fibrils (gray). The fibrils are elongated by further monomer addition (*k*_e_), and their surface catalyzes the formation of new aggregates via the process of secondary nucleation. Secondary nucleation is a two-step process involving binding of monomers to the fibril (*k*_b_), and their conversion to fibrils (*k*_2_).^45–47^ The surface of fibrils can be fully occupied by protein monomers at high-concentration regime, and their conversion into new fibrils becomes the rate-limiting step (red arrow in the right). This translates to a low monomer dependency of the aggregation. The scheme was inspired by Figure 3 in ref 46.

### Proteins glyB from HSV-1 and OspA/OspC from Bb Modulate the Aggregation of Aβ42 In Vitro with Different Potencies

To assess the interaction between selected proteins and Aβ42 (Table 1 and Figure 2a), we monitored its aggregation in the presence of increasing concentrations using the ThT assay (Figure 2b). We analyzed the resulting kinetic traces using the sigmoidal function (eq 1) and quantified the effects using changes in aggregation half-times (i.e., times to reach half-maximum signal) as a function of protein concentration (in molar equivalents, i.e., [protein]/[Aβ42], Figure 2c). Significant changes in Aβ42 aggregation were observed for three of the four proteins. The highest effect was observed for HSV-1 glycoprotein B (glyB), which significantly increased the lag time of Aβ42 aggregation in substoichiometric amounts. The protein was ThT positive in the absence of Aβ42, showing linear scaling of the signal with its concentration that was subtracted from the raw data during analysis (Supporting Information Figure S5a,b). The ThT fluorescence of the glyB alone remained linear during 40 h of incubation at 37 °C, which suggests that the protein did not aggregate during the time course of the experiment. Similarly, complementary experiments using static light scattering showed no evidence of a transition to higher-order assemblies. AFM analysis of the incubated sample confirmed that glyB did not form amyloid fibrils by itself (Supporting Information Figure S1), but revealed the presence of small, globular oligomers with a mean height of 4.8 nm. Based on these findings, along with complementary analyses of the fresh sample by DLS and mass spectrometry, we conclude that these oligomers likely arise from misfolding and represent a minor fraction of the total sample (Supporting Information Figure S1). Another control experiment confirmed that the storage buffer did not affect Aβ42 aggregation (Supporting Information Figure S5c).

**Figure 2:**
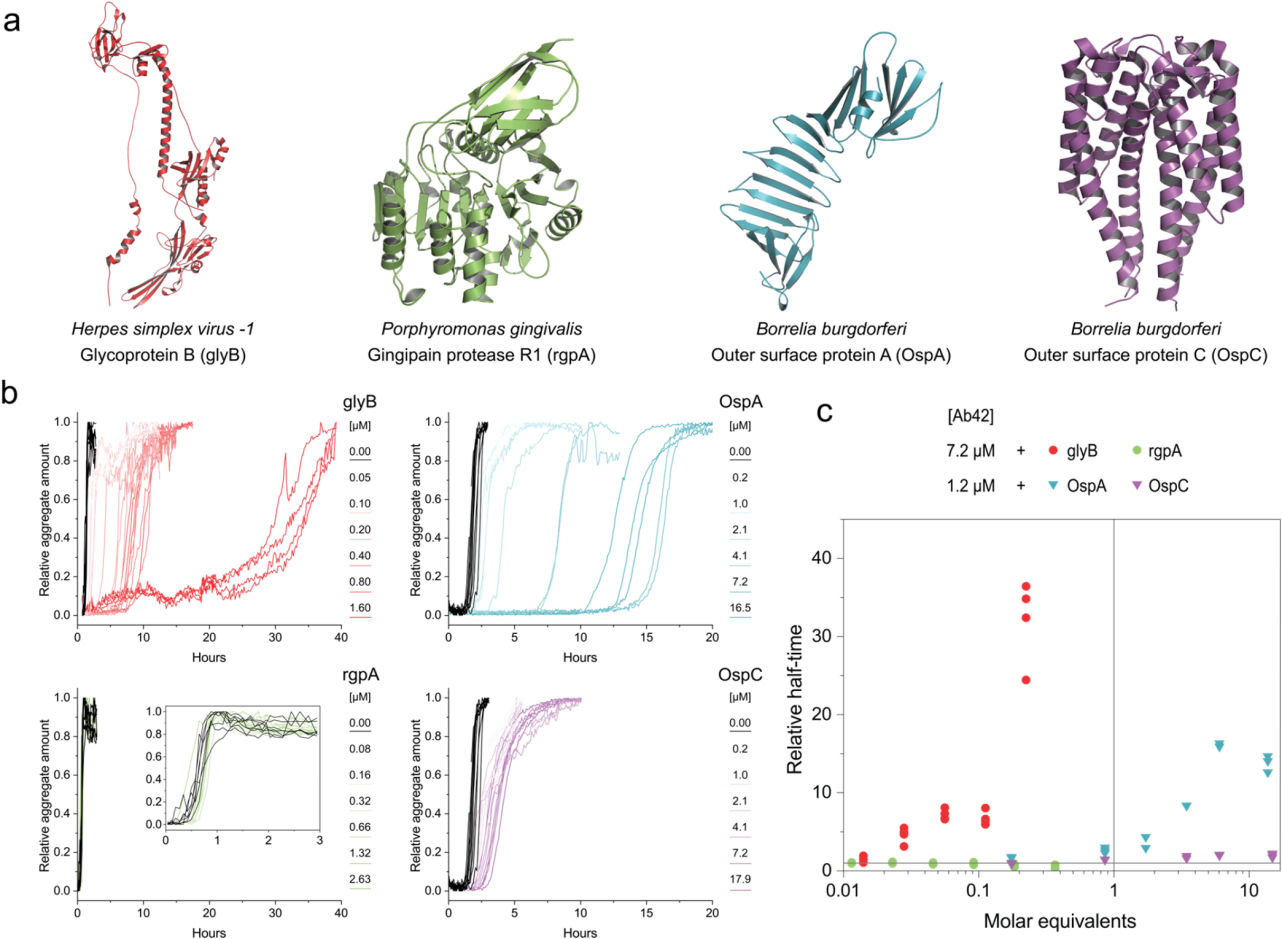
Aggregation of Aβ42 in the presence of proteins from pathogens associated with AD. (a) Overview of the protein structures used in this study. The 31–774 residue monomer fragment of glyB (red, PDB-ID:5v2s) used in this study is shown in red. The protein forms homotrimer on the surface of the virion. AlphaFold2 model of *P. gingivalis* rgpA (UniProt ID: P28784, residues 228–720) is shown in green. Structures of Bb outer surface proteins OspA (1osp) and OspC (1ggq) are shown in blue and violet, respectively. (b) Aggregation kinetics of Aβ42 in the presence of different amounts of glyB (red), rgpA (green), and OspA (blue) and OspC (violet). The aggregation of Aβ42 in the absence of any other protein is shown in black. The concentration of Aβ42 was kept constant at 7.2 μM (glyB, rgpA) and 1.2 μM (OspA, OspC) in each set of experiments. Each condition was carried out in 2–4 replicates. (c) Effect of proteins on the half-time of Aβ42 aggregation. The half-times obtained from independent fitting of each aggregation curve are plotted relative to those of Aβ42 alone (*t*_0.5_ (Aβ42+protein)/*t*_0.5_ (Aβ42)) as a function of protein concentration in terms of molar equivalents–[protein]/[Aβ42]. The points above the horizontal line at value 1 indicate an inhibitory effect.

Similarly to glyB, the presence of OspA resulted in a prolonged lag time of Aβ42 aggregation but only at higher molar equivalents of the protein and in experiments involving lower concentrations of Aβ42 (Figure 3). Indeed, neither OspA nor OspC showed inhibitory effects within the same experiment as glyB and rgpA (Figure 3, dark blue symbols). This suggests that OspA interacts weakly with the peptide compared with glyB or interferes with different aggregation pathways or species of Aβ42, as discussed further in the text. The effect of OspC was lower than that of OspA and could also be detected only at stoichiometric ratios and low Aβ42 concentration. Under these conditions, OspC mildly attenuated the Aβ42 lag time and slowed the rate of its aggregation (slope of the curve). Finally, we did not observe any influence of rgpA on Aβ42 aggregation (Figure 2b and Supporting Information Figure S5d–f). Based on the analysis, we conclude that surface proteins from HSV-1 and *Borrelia* interact with Aβ42 with different potencies, whereas soluble protease from *P. gingivalis* does not affect its aggregation.

**Figure 3:**
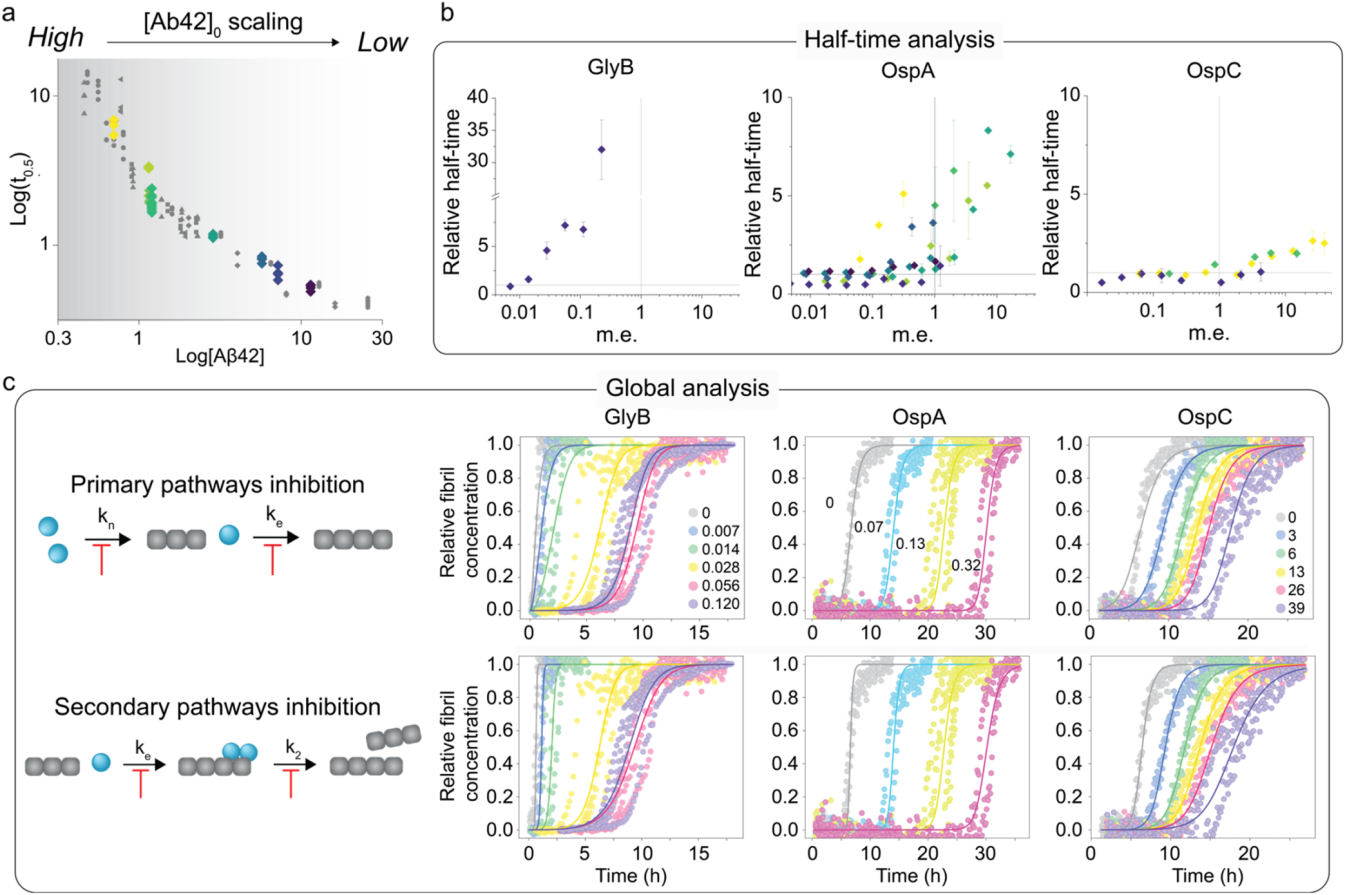
Mechanistic analysis of the protein’s effects on the aggregation of Aβ42. (a) Monomer scaling of Aβ42 aggregation. The different concentrations of Aβ42 that were used in experiments with glyB, OspA, and OspC are highlighted in color from the lowest (0.72 μM, yellow) to the highest (26 μM, dark blue). Gray to white background shading indicates the decreasing scale of the aggregation rate with monomer concentration. (b) Changes in half-times of Aβ42 aggregation as a function of different protein concentrations. The half-times obtained from independent fitting of each aggregation curve are plotted relative to those of Aβ42 alone (*t*_0.5_ (Aβ42+protein)/*t*_0.5_ (Aβ42)) as a function of protein concentration in terms of molar equivalents (i.e., m.e. = [protein]/[Aβ42]). The points are average values from 2 to 4 replicates of each condition with standard deviation. Symbol colors indicate the different concentrations of Aβ42 used in each of the experiments according to color-coding in A. (c) Global analysis of selected experimental data sets. For each experimental data set, global fitting to the model of multistep secondary nucleation was carried out using AmyloFit v2.0.^44^ For each case, inhibition of primary (*k_n_k*_e_) or secondary (*k*_2_ *k*_e_) pathways schematically shown on the left was probed by fitting one combined constant globally while allowing the other to vary for different protein concentrations. The data set of GlyB corresponds to the one in B ([Aβ42] = 7.2 μM). Data sets of OspA and OspC correspond to those whose half-times are indicated by the yellow diamonds in the middle and right graphs in B, respectively ([Aβ42] = 0.72 μM). Molar equivalents of the protein with respect to Aβ42 are indicated in the top plots.

### Kinetic Analysis Reveals Weak Interaction between Bb Outer Surface Proteins OspA and OspC and Monomeric Aβ42

An interesting observation from the initial screening of proteins’ influence on Aβ42 aggregation was that the inhibitory effect of OspA and OspC could only be observed in experiments in which the Aβ42 concentration was low (Figure 2b,c). We hypothesized that these effects correlate with the strength of interactions between the proteins and Aβ42 monomer. To test this, we repeated the kinetic experiments at different Aβ42 concentrations (Figure 3a). Altogether, we collected six experimental data sets with OspA, three with OspC, and one with glyB and rgpA owing to their limited availability. The inhibitory effect of both OspA and OspC decreases with increasing the Aβ42 concentration (Figure 3b). OspA significantly increased the half-time of Aβ42 aggregation below 2 μM (yellow and green symbols in the middle graph of Figure 3b). At higher concentrations, reduced rate and prolonged lag time could still be observed, albeit at lower magnitudes. A similar trend was observed for OspC, which required higher molar equivalents for a significant effect. The concentration range in which both OspA and OspC switch from strong- to low-influence on Aβ42 correlates with the change of the rate-limiting step (i.e., monomer scaling) of Aβ42 aggregation (Figure 1c). Decreased monomer dependency due to the saturation of secondary nucleation at high concentrations of Aβ42 combined with the lack of effect observed for OspA and OspC (and for rgpA) at this regime is a strong indication that the two proteins interact directly with monomeric Aβ42. This is further supported by the stoichiometric ratios at which these proteins affect Aβ42 aggregation, with OspA showing a stronger interaction than that of OspC at low Aβ42 concentrations.

Furthermore, we analyzed the effects of glyB, OspA, and OspC by global analysis using the microscopic aggregation framework for Aβ42 described earlier in the text (Figure 1). Specifically, we fitted each data set globally to the multistep secondary nucleation model using AmyloFit v2.0 (Figure 3c). In each case, we modeled scenarios in which the proteins inhibit either primary (combined rate constant of primary nucleation and elongation—*k_n_k*_e_) or secondary (combined rate constant of secondary nucleation and elongation—*k_n_k*_e_) pathways by fitting one combined constant globally while allowing the other to vary for different protein concentrations. Representative fits are shown in Figure 3c. In all cases, both scenarios explain the data reasonably well. However, we observe differences between OspA and OspC upon a closer investigation of the quality of the fits (correspondence of the data and shape of the fitted curves). OspA induces a significant shift of Aβ42 aggregation lag time without affecting the slope of the curves, which is consistent with inhibition of primary nucleation. Conversely, the effect of OspC on the kinetic slopes is greater than that of lag times, and a better fit is obtained for the inhibition of secondary pathways. Importantly, the lack of inhibition in experiments with high Aβ42 concentrations further supports our hypothesis that OspA and OspC interact with monomeric (or other soluble) species of Aβ42. In the case of glyB, inhibition of either primary or secondary nucleation provides good fits, with the latter being slightly better in terms of the mean squared error. The effect on the lag time indicates interaction with soluble Aβ42, however, interaction with Aβ42 fibrils cannot be excluded based on our data set. Additional experiments need to be carried out in the future to further delineate what oligomeric species primarily interact with Aβ42.

### Effects of HSV-1 Infection and Recombinant OspA/OspC Treatment on APP Accumulation and Aβ40/Aβ42 Secretion in Human Neuronal Models

To support the findings from *in vitro* experiments using human-relevant cellular models, we utilized human neurons derived from two lines of human pluripotent stem cells: ESI-017 hESC-derived neural stem cells: (i) NSCs^48^ and (ii) i3N-iPSCs.^49^ Following a 14-day differentiation period, the neurons were either infected with HSV-1 or treated with recombinant OspA or OspC. Initially, we aimed to assess the extent of APP accumulation in 2D neuronal cultures and conducted immunohistochemistry, followed by image analysis. The analysis involved determining the total amount of APP signal per cell nucleus and evaluating the percentage of large APP particles per sample, which provided insights into the amount of APP clusters in the 2D neurons. As depicted in Figure 4a,b and quantified in Figure 4c,d, our TUJ-positive neuronal cultures naturally secreted APP protein and exhibited a basal level of APP clustering. Importantly, compared to the control, HSV-1 infection led to a significant increase in the amount of APP signal (Figure 4b,c) and a significantly higher percentage of large APP clusters (Figure 4d). On the other hand, treatment with OspA resulted in a moderate increase in APP signal, accompanied by a trend toward APP clustering, although this trend did not reach statistical significance (Figure 4b–d). Treatment with OspC showed even milder changes, with lower APP expression and clustering observed (Figure 4b–d). To confirm the specificity of the amyloid antibody (D54D2), we conducted a control experiment using Vero cells, which lack endogenous APP, and confirmed that the antibody does not bind HSV-1 nonspecifically (Supporting Information Figure S6).

**Figure 4:**
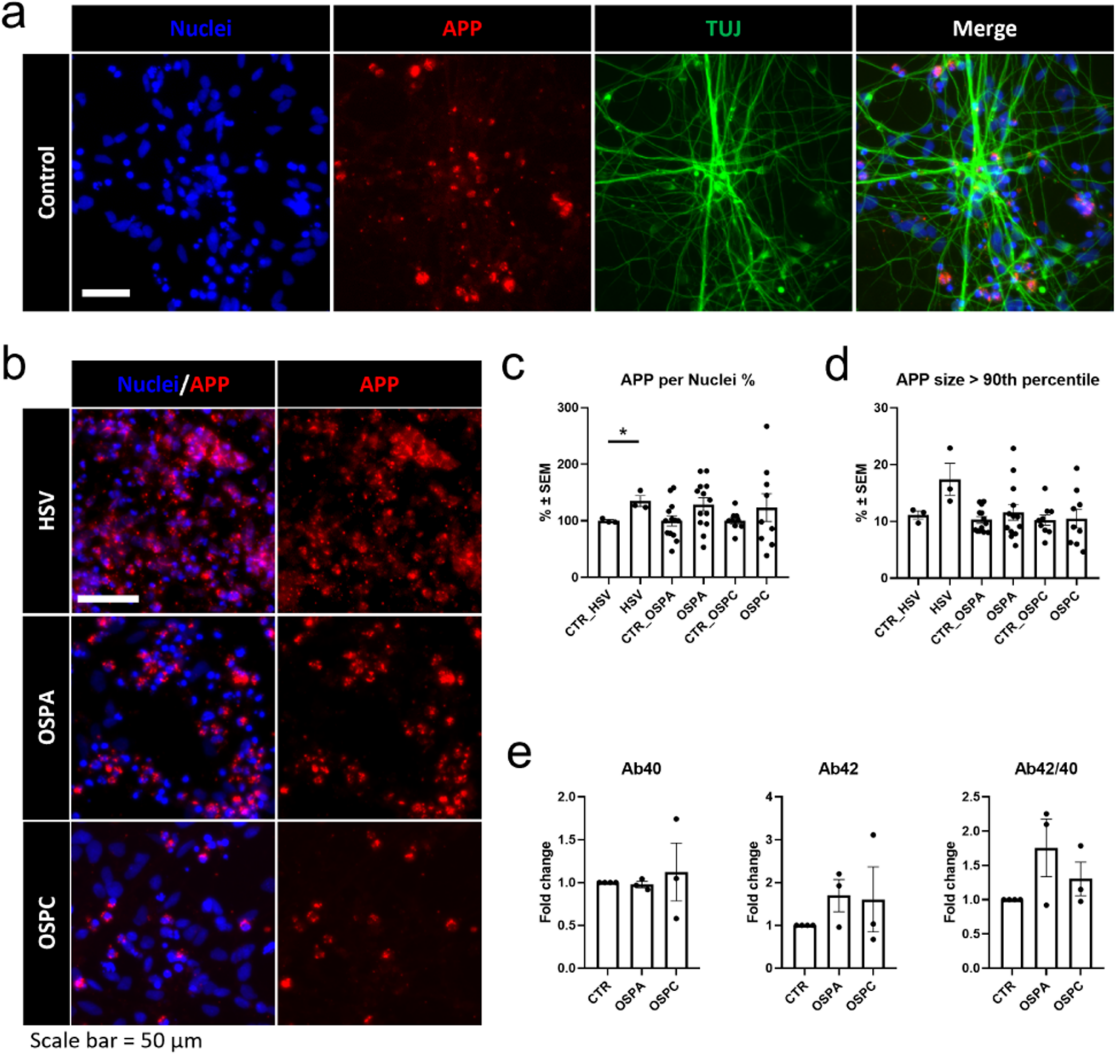
Effects of HSV-1 infection and recombinant OspA/OspC treatment on APP accumulation and Aβ secretion in human neuronal models. (a) Immunocytochemistry of TUJ-positive neurons and APP signal on untreated human pluripotent stem cells-derived neurons. Nuclei were counterstained with Hoechst. Scale bar: 50 μm. (b) APP signal visualization of neuronal cultures after infection with HSV-1 and treatment with OspA or OspC. Nuclei were counterstained with Hoechst. Scale bars: 50 μm. (c, d) Quantification of the APP signal in acquired images. Visualized is the (c) amount of APP signal per nucleus and (d) the size of APP clusters visualized as the 90th percentile of individual APP clusters in three or more replicates. *p < 0.05. (e) Amount of secreted Aβ40 and Aβ42 peptides in the cell culture media, and the Aβ42/40 ratio measured by ELISA. Values represent pg of Aβ/μg of protein in three or more replicates. Error bars represent ± SEM.

Additionally, since the results from OspA and OspC treatment showed an apparent trend in APP accumulation compared to the strong cellular response induced by HSV-1 infection, we investigated whether OspA and OspC treatments stimulated the secretion of Aβ40 and Aβ42 peptides, which were shown to exhibit antimicrobial activity in *in vivo* and *in vitro* models.^50,51^ We also evaluated the ratio of Aβ42/40 in the secreted neuronal cultures, as it serves as an indicator of ongoing AD-like pathology in *in vitro* neuronal models, as previously shown by us and others^52^ and reviewed in ref 53. Remarkably, our ELISA measurements supported the trends observed in the immunohistochemistry results, where OspA treatment with βspA led to a moderate increase in the secretion of Aβ42, while OspC treatment with βspC resulted in a milder increase in Aβ42 secretion (Figure 4e). The evaluation of the Aβ42/40 ratio again showed an increase in trend upon treatment (Figure 4e). These results confirm the *in vitro* data, demonstrating the diminishing affinity of recombinant Aβ to selected recombinant proteins from HSV-1 and Bb.

## Discussion

One of the strong appeals of the infectious hypothesis of AD is a finding that Aβ42 acts as an antimicrobial peptide (AMP).^50,51,54^ In the key study, Aβ42 exerted antimicrobial activity to eight common pathogens with similar or, in some cases, even greater potency to LL-37, a known human AMP showing conformational similarities to Aβ42.^50^ Furthermore, it has been demonstrated that aggregation of Aβ42 is seeded by the HSV-1 virus, which prevented its entry into the host cells by entrapment and agglutination of the viral particles.^54–57^ Although currently, no definite evidence or causal relationship between infection and AD exists,^58^ these and other studies point out the potential physiological role of Aβ42 in the brain’s innate immunity. Here, we investigated the effects of four different proteins, from pathogens associated with AD to the aggregation of Aβ42. We used kinetic analysis, which has been previously successfully applied to access the effects and mechanisms of small-molecule inhibitors,^59^ chaperones,^60^ or antibodies^61^ to Aβ aggregation. Although the pathogen hypothesis of AD was proposed more than 100 years ago,^62–64^ only a handful of studies currently study the effect of proteins from pathogens associated with AD within the kinetic analysis network of Aβ aggregation.^65^

Our results are in correspondence with the previously described interaction between Aβ and HSV-1 glycoprotein B^20,54^ and newly show similar (albeit weaker) effects of outer surface proteins OspA and OspC from *B. burgdorferi*. Conversely, we observed no effect of the soluble protease rgpA on Aβ aggregation in our ThT assay. It was shown previously that *P. gingivalis* gingipains have cathepsin B-like activity, and their mechanism of action is likely linked to increased Aβ production via its cleavage from APP.^10,66^ It has been demonstrated that Aβ42 binds HSV-1 via its surface glycoproteins, which triggers its aggregation, leading to agglutination and entrapment of the viral particles.^54^ Here, we observe attenuation of Aβ42 aggregation in the presence of a soluble construct of HSV-1 glycoprotein B due to its interaction with soluble Aβ42. We hypothesize that these seemingly contrasting effects can be explained by the different contexts of the protein from pathogens, i.e., soluble versus surface-bound. The binding of Aβ42 to glyB displayed on the surface of HSV-1 leads to an increase in its local concentration and formation of fibrils via heterogeneous, surface-catalyzed nucleation.^67^ Alternatively, the distinct effects can be attributed to the different conformations of glyB in the membrane-bound and free states. Based on our data, we cannot distinguish the primary species of glyB that interacts with Aβ42. Additional experiments involving smaller fragments such as individual domains or peptides derived from the proteolytic processing of glyB by host enzymes are needed to provide more detailed mechanistic insights into the interaction between Aβ42 and glyB described here.

Similar context-dependent effects can be arguably expected for outer surface proteins of *B. burgdorferi*, leading to agglutination and entrapment of the bacterium *in vivo*, as observed for *Candida albicans* or *Salmonella typhimurium* in model experiments.^51^ To simulate the effect of the surface, we repeated the Aβ42 aggregation experiment in the presence of OspA covalently immobilized onto beaded agarose resin (Supporting Information Figure S7). Interestingly, we observed an acceleration of Aβ42 aggregation induced by the presence of immobilized OspA (Supporting Information Figure S7), supporting our hypothesis about the role of surfaces. However, further studies need to be carried out to fully elucidate the exact mechanism. *In vivo*, Aβ seeding by pathogen surface is likely a multistep process that might involve more proteins, protein fragments, or even nonprotein moieties such as carbohydrates. Here, we demonstrate that kinetic analysis is a valuable tool for screening potential interaction partners from pathogens in a relatively simple and high-throughput manner. In the future, it can be extended to screening effects of other glycoproteins, capsid, and tegument proteins of HSV-1 or their fragments that could potentially interact with Aβ or APP and modulate its aggregation. Amyloidogenic regions have been identified and experimentally described in other viruses, demonstrating the rich amyloid landscape of viral proteins.^68,69^ Their exposure by cleavage by host proteases may shift the balance between Aβ production, accumulation, and clearance toward pathological states. Nonproteinaceous pathogen materials such as extracellular DNA, lipopolysaccharides, proteoglycans, or carbohydrates can induce similar effects, highlighting the variety of pathogen-associated factors that can influence Aβ aggregation and potentially contribute to neurodegeneration.^54,70–72^

We further extended our study of proteins from pathogens by experiments with stem cell-derived neurons. Previous research has demonstrated that pathogen infections can induce Aβ oligomerization and accelerate β-amyloid deposition in both cellular and animal models.^50,51^ While many studies focus on the effects of HSV-1 on Aβ/APP production and accumulation in different *in vivo* and *in vitro* models (e.g., mice, worms, human neuroglioma (H4) cells, and Chinese hamster ovary (CHO) cells), the impact of *B. burgdorferi* or its outer surface proteins remains unexplored despite its reported association with AD. This is likely due to challenges associated with Bb *in vitro* culture. Here, we show that in addition to the propensity of OspA and OspC toward modulation of Aβ aggregation *in vitro*, cultured human neurons also react to Bb outer protein OspA, exhibiting increased Aβ42 secretion and formation of larger APP clusters.

## Conclusions

In summary, this study examined how proteins from pathogens associated with AD affect the aggregation of Aβ42. We found that in their soluble form, HSV-1 glycoprotein B and *B. burgdorferi* outer surface proteins OspA and OspC interact with Aβ42 and attenuate its aggregation. Conversely, the protein OspA accelerates aggregation when immobilized on a surface, suggesting that surface proteins may recognize Aβ42 and trigger its aggregation via surface-catalyzed nucleation. Experiments with stem cell-derived neurons showed that Bb outer protein OspA mildly increases Aβ42 secretion and induces the formation of larger APP clusters. The study underscores the complex role of proteins from pathogens in AD and highlights kinetic analysis as a valuable tool for both preliminary screening and in-depth mechanistic research.

## Materials and Methods

### Protein Preparation

Recombinant virion surface domain of the HHV-1 envelope glycoprotein B (glyB) corresponding to residues 31–774 of the full-length protein (UniProt ID P08665) and recombinant *P. gingivalis* Gingipain R1 (rgpA) corresponding to residues 228–720 of the full-length protein (P28784) were purchased from MyBioSource (San Diego, USA). Both proteins were expressed in mammalian cells and purified via *N*-terminal His-tag 6x by the company.

Plasmids for recombinant expression of *Borrelia burgdorferi* *sensu lato* outer surface proteins A (OspA) and C (OspC) were kind gifts from Prof. Milan Raska (Faculty of Medicine, Palacky University Olomouc) and Dr. Adam Norek (CEITEC, Brno), respectively. *N*-terminal signal peptide of OspA was replaced by a His-tag followed by a 16-residue linker, an enterokinase cleavage site, and an OspA sequence corresponding to residues 17–273 of the full-length protein (UniProt ID B7IZU3). *N*-Terminal signal peptide of the OspC was replaced by C-terminally his-tagged polyubiquitin-B (UniProt ID J3QS39), followed by TEV protease cleavage site and the OspC sequence corresponding to residues 11–191 (UniProt ID P94245). Genes were expressed in *E. coli* BL21 (DE3) cells under the control of the T7 promoter and ampicillin or kanamycin as a selection marker for OspA and OspC, respectively. Proteins were purified by affinity chromatography via a His-tag, which was subsequently cleaved by the respective protease and removed by a second round of affinity chromatography using TALON Superflow (Cytiva, Marlborough). Monomer and dimer of OspA and OspC, respectively, were isolated by size exclusion chromatography using HiLoad 16/600 superdex 75 pg column (Cytiva, Marlborough) equilibrated with either PBS or 20 mM sodium phosphate with 200 μM EDTA pH 8 for cell culture experiments and in vitro assays, respectively.

### Expression and Purification of Recombinant Aβ42

Amyloid β 42 with *N*-terminal methionine (Aβ42) used in this study was expressed recombinantly from *E. coli* and purified from inclusion bodies according to published protocols.^41,42^ Shortly, Aβ42 was expressed to inclusion bodies in *E. coli* BL21 (DE3) cells at 37 °C. Inclusion bodies were isolated by three cycles of sonication, centrifugation, and resuspension followed by dissolution in 8 M urea. Purification of Aβ42 from inclusion bodies was carried out by ion exchange chromatography using DEAE Sepharose Fast Flow (Cytiva, Marlborough, MA) in batch mode. Fractions eluted by 10 mM Tris-HCl and 1 mM EDTA pH 8 with increasing concentration of NaCl (0–100) were applied onto the HiLoad 16/600 superdex 75 pg column, and the peak fraction corresponding to Aβ42 was collected, split into three identical aliquots, and lyophilized. Fresh, monomeric Aβ42 was always prepared prior to the experiment by size exclusion chromatography of the peptide dissolved in 6 M GndHCl using a Superdex 75 10/300 GL column equilibrated with 20 mM sodium phosphate and 200 μM EDTA, pH 8. The center of the elution peak was collected into a chilled LoBind microcentrifuge tube (Fisher Scientific, Waltham, MA) on ice and used immediately. For each measurement, fresh stock of monomeric Aβ42 was isolated by size exclusion and used immediately (within hours) while handled on ice. The concentration of Aβ42 was determined spectrophotometrically using a calculated extinction coefficient ε_280_ = 1490 M^–1^ cm^–1^.

Sequences of all proteins used in this study are provided in the Supporting Information.

### Biophysical Characterization of the OspA and OspC

Purity of the samples (>95%) was verified by SDS-PAGE. Correct folding was assessed by near-UV circular dichroism (CD) spectroscopy using a Chirascan spectrophotometer (Applied Photophysics, Leatherhead, UK). Spectra were collected between 185 and 260 at 1 nm bandwidth and 0.5 s integration time in 5 replicates. Buffer spectrum was subtracted from the averaged sample spectra. Potential aggregation of the proteins was probed by monitoring the static light scattering signal (SLS) at 266 nm at 37 °C over the period of 2 days using UNcle instrument (Unchained Laboratories, Pleasanton). Sample concentration was varied from 0.01 to 25 μM.

### Biophysical Characterization of glyB

#### Dynamic and Static Light Scattering Analysis

The purity of the samples was confirmed to be >85% by the supplier (MyBioSource) based on the SDS-PAGE analysis. Stock protein was diluted to the assay buffer (20 mM sodium phosphate 200 μM EDTA pH 8), and the oligomeric size distribution of samples at 0.25, 0.5, 1, and 2 μM was carried out by DLS using Prometheus Panta (NanoTemper, Germany). Samples were loaded into glass capillary in duplicates, and autocorrelation curves were measured in ten 5-s acquisitions per capillary. The size distribution was derived from the average of the measurements automatically by PR.PantaAnalysis software (NanoTemper, Germany) using the size distribution fit. Static and dynamic light scattering of the same samples was monitored at 37 °C for 40 h using a Prometheus Panta.

#### AFM Analysis of glyB after Incubation

The 2 μM glyB sample incubated at 37 °C for 40 h was deposited onto freshly cleaved mica substrates. Following 2 min of incubation, the substrate was washed extensively with Milli-Q water and dried under nitrogen gas flow. The sample was imaged in tapping mode in air by DriveAFM (Nanosurf, Liestal, Switzerland) using PPP-NCLAuD cantilevers (Nanosensors, Neuchatel, Switzerland). The oligomer heights were derived from profiles of manually selected particles from a representative region of interest using Gwyddion software.

#### Mass Photometry Analysis of glyB Oligomeric States

The mass photometry experiments of glyB were carried out using a TwoMP instrument (Refeyn Ltd.). Stock of 1 μM glyB was prepared by dilution of the stock to the assay buffer (20 mM sodium phosphate, 200 μM EDTA pH 8). The ratiometric contrasts of individual particles were recorded automatically for glyB samples in the 18.77 to 300 nM protein concentration range during 1 min acquisition directly upon dilution to the buffer from the stock. The ratiometric contrast values were recalculated to the molecular weights using a calibration curve made using bovine serum albumin (BSA), Immunoglobulin G (IgG), and Thyroglobulin (TG) samples of known molecular mass. The relative fraction of monomer, dimer, trimer, and low-molecular-weight oligomers was calculated for each acquisition as a fraction of counts in the 0–300 and 400–800 kDa ranges, respectively, divided by the total counts. Each experiment was carried out in duplicate.

### Thioflavin T Aggregation Assay

Thioflavin T (ThT) assay was used to monitor the aggregation kinetics of Aβ42 alone or in the presence of proteins of interest (POI). Buffer, protein, and Aβ42 solutions were supplemented by ThT (15 μM) from a 3 mM ThT stock and mixed to desired final concentrations and ratios. All mixing was done on ice, and Aβ42 was always added as the last component. Measurements were carried out using a Synergy H4 hybrid microplate reader (Fisher Scientific, Waltham) in the Corning 384-well Black/Clear Flat Bottom Polystyrene NBS Microplates (Corning, NY) sealed with sealing tape (Corning, NY). The instrument was pre-equilibrated to the assay temperature (37 °C), and the kinetics of Aβ42 aggregation was monitored by the changes in ThT fluorescence (ex.440/em.485 nm) over time under quiescent conditions. Experiments were carried out in triplicate or quadruplicate with 40 μL of solution per well.

Each kinetic trace was fitted individually by a four-parameter sigmoidal curve (eq 1).^73^

1 $$F\left(t\right)=F_{0}+A/\left(1+\exp\left(-k\left(t-t_{0.5}\right)\right)\right)$$

Fitted parameters are the initial baseline fluorescence, *F*_0_, the amplitude, *A*, the aggregation rate constant, *k*, and the time at half completion of the aggregation, i.e., half-time, *t*_0.5_. The lag time of the aggregation was calculated from the fitted parameters according to

2 $$t_{\mathrm{lag}}=t_{0.5}-2/k$$

### AFM Imaging

The visualization of the reaction species during or after the aggregation of the Aβ42 with or without POI was carried out using Bruker Dimension FastScan AFM equipped with a Bruker SCANASYST-FLUID+ probe (both Bruker Nano Surfaces, Tucson). Sensitivity and cantilever spring constant were calibrated by the routine procedure recommended by the instrument manufacturer prior to each measurement. Freshly prepared Aβ42 was mixed with different concentrations of OspA, with OspC, or with buffer and incubated at 37 °C in the low-binding Eppendorf tubes. Aliquots of 20 μL were withdrawn from the reaction mixture at fixed time intervals and spotted onto the freshly cleaved silanized ((3-Aminopropyl)­trimethoxysilane) mica surface. Samples were incubated for 20 min and washed 3 times with 200 μL of buffer or deionized water. Images covering an area of 10 × 10 μm^2^ were recorded with a set point of 0.75 nN and a lifting height of 150 nm. For each condition/time point, 4 images of randomly selected areas were acquired. The probe height images were processed in Gwyddion software by the removal of the polynomial background.

### OspA Immobilization

Sulfolink coupling resin (Thermo Fisher Scientific, Waltham) was used for OspA immobilization according to the manufacturer’s protocol. Resin was equilibrated by coupling buffer (50 mM Tris, 5 mM EDTA-Na; pH 8.5) and distributed equally (0.5 mL resin bed volume) to four gravity columns. Stock solution of OspA (22 μM) was diluted two and four times by coupling buffer and supplemented with TCEP (25 mM). Each of the three OspA solutions and the buffer (negative control) was then mixed with the resins in the columns. Coupling was carried out at RT for 60 min, followed by blocking of the unreacted iodoacetyl groups by L-cysteine, washing 3 times with 1 M NaCl, and equilibrated to 20 mM sodium phosphate and 200 μM EDTA pH 8 for ThT assay. Coupling efficiency (78%) was determined as the ratio between protein concentration in solution before coupling and flow through from the first wash step. Dilution series (1–16-fold) of each of the four solutions was prepared and mixed with freshly prepared Aβ42, supplemented with ThT (15 μM), and the fluorescence intensity at 485 nm upon excitation at 440 nm was monitored under slow shake conditions at 37 °C.

### Cell Culture and Differentiation

All experiments were performed on human pluripotent stem cell-derived neurons differentiated from two independent cell lines, i3N-iPSCs^49^ and ESI-017-NSCs.^48^ i3N-iPSCs were maintained and differentiated as described previously in ref 49,74 . Briefly, feeder-free cultures of i3N-iPSCs were grown on Matrigel-coated plates (Corning) in mTeSR1 (STEMCELL Technologies) and passaged using 0.5 mM EDTA (Thermo Fisher Scientific) in PBS. Neuronal differentiation was induced using the overexpression of Neurogenin 2 (NGN2) via Doxycycline for 3 days, as described in ref 49. Cells were then replated on glass coverslips coated with poly-l-ornithine and laminin (Thermo Fisher Scientific) and maintained in a Differentiation medium containing BrainPhys Neuronal Medium (STEMCELL Technologies), B27 medium supplemented with vitamin A (Thermo Fisher Scientific), NT3 (Peprotech), BDNF (Peprotech), and laminin for another 14 days. Subsequently, neurons were treated with HSV-1, OspA, or OspC, as described below.

As a second cell line, established self-renewing neural stem cells (CoMo-NSCs) were derived from human embryonic stem cells (cell line ESI-017, ESI BIO, Alameda, CA) and cultured as described previously.^75^ Briefly, NSCs were maintained on cell culture plates coated with poly-l-ornithine and laminin using the NSC Growth medium containing DMEM/F12, 1% Glutamax, 1% nonessential amino acids, 0.5% N2 supplement, 0.5% B27 supplement without vitamin A, recombinant human fibroblast growth factor 2 (FGF2) (Thermo Fisher Scientific), and Zell shield cell culture contamination preventive solution (Minerva Biolabs) at a concentration of 5 μL/mL. Cells were passaged using Accutase (Thermo Fisher Scientific). For the induction of terminal differentiation, NSCs were seeded on day 0 at a density of 25,000/cm^2^ on 24-well plates with coverslips and incubated at 37 °C with 5% CO_2_ in the NSC Growth medium with FGF2. Starting from day 3, cells underwent differentiation in the NSC Growth medium without FGF2, supplemented with 5 μL/mL of the Zell shield solution. The medium was changed every other day for 14 days. Subsequently, neurons were then treated with HSV-1, OspA, or OspC as described below.

### Infection of Neurons with HSV-1

Mature human neurons were infected with HSV-1 viral particles (strain McIntire, kindly provided by Prof. Andreas Sauerbrei, German Reference Laboratory of HSV and VZV, Germany) cultured in Vero cells (ATCC CCL-81, African Green Monkey, adult kidney, epithelial) as described previously in ref 76. Specifically, HSV-1 viral particles at a multiplicity of infection (MOI) of 0.0001 were resuspended in a viral cell culture medium containing DMEM (Sigma-Aldrich) and 10% fetal bovine serum (Sigma-Aldrich), 1% penicillin-streptomycin (Sigma-Aldrich), and 1% l-glutamine (Thermo Fisher Scientific). Subsequently, 0.5 mL of this medium containing HSV-1 was added directly to the culture of human neurons for a duration of 30 min. Following the infection period, the neuronal cell cultures were rinsed with fresh neuronal cell culture media and maintained under standard neuronal cell culture conditions for 4 days. Following this incubation, the cells were fixed by using a 4% Paraformaldehyde solution and subjected to immunocytochemical processing. As a control group, neurons treated with a Viral cell culture medium without HSV-1 were used.

### Cell Culture and Infection of Vero Cells

Vero cells (derived from African green monkey kidney) were used to assess the specificity of β-amyloid (APP) antibody binding (Supporting Information Figure S6). Cells were seeded at a density of 20,000 per well in a Greiner 96-well plate. The following day, they were infected with HSV-1 (MacIntyre strain) at a multiplicity of infection (MOI) of 1 and incubated for 24 h. After infection, cells were fixed and immunostained using the following marker combinations: β-amyloid/ICP4, β-amyloid/HSV-1, and DAPI for nuclear staining.

### Treatment of Neurons with OspA and OspC

Mature human neurons were treated with recombinant proteins prepared as described above. For cell culture experiments, the solution of a solution of an OspA or an OspC prepared in PBS was diluted to a final concentration of 10 μM in a neuronal cell culture medium. Subsequently, 0.5 mL of medium containing PBS and OspA or OspC was added directly to the culture for human neurons. Half of the medium in the wells was changed every 2 days for a new one containing freshly added OspA or OspC. For immunocytochemistry, cells were fixed using a 4% Paraformaldehyde solution after 7 days and then subjected to immunocytochemical processing. For ELISA, cells were rinsed once, and the medium on day 7 was changed to Essential 6 Medium (Thermo Fisher Scientific). After an additional 3 days *in vitro* without any medium change, the medium was aspirated and stored at −80 °C until processing. Cells from the same wells were also lysed in 1% SDS lysis buffer 9 ^77^ and used to normalize data from ELISA. As a control group, neurons treated with a medium with PBS but without OspA or OspC were used.

### Immunocytochemistry, Microscopy, and Image Analysis

Immunocytochemistry of neurons was performed as described previously.^78^ Briefly, cells were fixed with 4% paraformaldehyde, permeabilized using 0.2% Triton ×100 in 1× PBS for 15 min, and incubated with primary antibodies overnight at 4 °C. Secondary antibodies and Hoechst were diluted in the permeabilization buffer and incubated with cells for 1 h at room temperature. After incubation, the slides were washed carefully with PBS, dried, and mounted onto microscopic slides with Mowiol Reagent (Merck). The following primary and secondary antibodies were used: β-amyloid (rabbit, D54D2, Cell Signaling), β3-Tubulin TU-20 (mouse, 4466, Cell Signaling), donkey anti-Rabbit AF488 (A-21206, Invitrogen), and donkey anti-Mouse AF568 (A-10037, Invitrogen).

Samples were imaged with the widefield microscope Zeiss Axio Imager.Z2, equipped with a halogen lamp and N-Achroplan 20*x*/0.45 AIR objective using ZEN Blue software (Zeiss). Hoechst was detected using a 387/425 nm excitation filter, 446/468 nm emission filter, and 435 dichroic mirror. GFP was detected using a 450/490 nm excitation filter, 500/550 nm emission filter, and 495 dichroic mirror. Texas Red was detected using a 540/580 nm excitation filter, 593/668 nm emission filter, and 585 dichroic mirror. Images with a 0.65 × 0.65 × 1.53 μm pixel size were acquired using a monochromatic camera, Hamamatsu ORCA Fusion (sCMOS sensor). A total of 56 tiles were acquired from each sample and subsequently analyzed for APP accumulation.

To evaluate APP accumulation in human neurons, we used commercially available Imaris version 9.8.2 (Bitplane, South Windsor, CT). Detection of individual APP plaques was performed in Imaris by using the “Surface” module. The estimated parameters included the volume of the APP and nuclei. Parameters were automatically quantified using Imaris software. Data were analyzed and plotted using GraphPad Prism version 8.

### ELISA

For ELISA, individual wells with neurons treated with OspA or OspC were cultivated in Essential 6 Medium for 72 h before analysis. The cell culture medium and the corresponding neurons were collected and stored separately at −80 °C. The amount of Aβ40 and Aβ42 peptides in the cell culture media was measured with amyloid β 40 Human ELISA Kit (Thermo Fisher Scientific) and amyloid β 42 Human ELISA Kit, Ultrasensitive (Thermo Fisher Scientific) according to the manufacturer’s instructions. Samples were analyzed in technical duplicates. To compare the amounts of Aβ40 and Aβ42 peptides from different wells, we measured the total protein concentration of lysed neurons per well, calculated the total protein weight, and used it for normalization. Values represent picograms of Aβ/μg of protein.

### Quantification and Statistical Analysis of Cell Culture Experiments

Data analyses were done using GraphPad Prism version 8. A two-tailed Student’s *t* test was performed, and differences were considered statistically significant at *p < 0.05. All data are presented as mean ± SEM and plotted as a bar graph with depicted individual values as dots.

## Supplementary Material



## Notes

Supplementary figures and tables showing biophysical characterization of the proteins, AFM analysis of amyloid-β fibrils, and control aggregation and *in vivo* experiments (PDF)

## ∇

The current affiliation of the author is Protein Biophysics Group, Department of Biotechnology and Biomedicine, Technical University of Denmark, Søltofts Plads, Building 227, Kgs. Lyngby 2800, Denmark
