# Supplementary material for: Modulation of Amyloid‑β Aggregation by Surface Proteins from Pathogens Associated with Alzheimer’s Disease
Source: ACS Chem Neurosci. 2025 Aug 27;16(18):3554–66. doi: 10.1021/acschemneuro.5c00444 (PMC12447514; doi:10.1021/acschemneuro.5c00444)
Supplement: Supplementary file 1 [file cn5c00444_si_001.pdf]

SUPPLEMENTARY INFORMATION

Modulation of Amyloid-beta Aggregation by Surface Proteins from Pathogens Associated with Alzheimer's Disease

Antonin Kunka [1,2,§,], Hana Hribkova [3,*], Tereza Vanova [2,3], Veronika Pospisilova [3], Martin Havlasek [1,2], Jan Haviernik [4], Daniel Ruzek [4,5,6], Jiri Damborsky [1,2], Dasa Bohaciakova [2,3,#], Zbynek Prokop [1,2,#]*

1 Loschmidt Laboratories, Department of Experimental Biology and RECETOX, Faculty of Science, Masaryk University, Brno 625 00, Czech Republic

2 International Clinical Research Center, St. Anne's University Hospital Brno, Brno 602 00, Czech Republic

3 Department of Histology and Embryology, Faculty of Medicine, Masaryk University, Brno 625 00, Czech Republic

4 Veterinary Research Institute, Brno 621 00, Czech Republic

5 Department of Experimental Biology, Faculty of Science, Masaryk University, Brno 625 00, Czech Republic

6 Institute of Parasitology, Biology Centre of the Czech Academy of Sciences, Ceske Budejovice 370 05, Czech Republic

# To whom correspondence should be addressed. zbynek@chemi.muni.cz, bohaciakova@med.muni.cz

* The authors wish it to be known that, in their opinion, the first two authors should be regarded as joint First Authors.

[§] The current affiliation of the author is: Protein Biophysics Group, Department of Biotechnology and Biomedicine, Technical University of Denmark, Søltofts Plads, Building 227, 2800, Kgs. Lyngby, Denmark

Primary sequences of proteins used in this study:

Recombinant amyloid-beta 42

MDAEFRHDSGYEVHHQKLVFFAEDVGSNKGAIIGLMVGGVVIA

Glycoprotein B (HSV-1) – residues 31-774

APSSPGTPGVAAATQAANGGPATPAPPALGAAPTGDPKPKKNKKPKNPTPPRPAGDNATVAAGH
ATLREHLRDIKAENTDANFYVCPPPTGATVVQFEQPRRCPTRPEGQNYTEGIAVVFKENIAPYK
FKATMYYKDVTVSQVWFGHRYSQFMGIFEDRAPVPFEEVIDKINAKGVCRSTAKYVRNNLETTA
FHRDDHETDMELKPANAATRTSRGWHTTDLKYNPSRVEAFHRYGTTVNCIVEEVDARSVYPYDE
FVLATGDFVYMSPFYGYREGSHTEHTSYAADRFKQVDGFYARDLTTKARATAPTTRNLLTTPKF
TVAWDWVPKRPSVCTMTKWQEVDEMLRSEYGGSFRFSSDAISTTFTTNLTEYPLSRVDLGDCIG
KDARDAMDRIFARRYNATHIKVGQPQYYLANGGFLIAYQPLLSNTLAELYVREHLREQSRKPPN
PTPPPPGASANASVERIKTTSSIEFARLQFTYNHIQRHVNDMLGRVAIAWCELQNHELTLWNEA
RKLNPNAIASATVGRRVSARMLGDVMAVSTCVPVAADNVIVQNSMRISSRPGACYSRPLVSFRY
EDQGPLVEGQLGENNELRLTRDAIEPCTVGHRRYFTFGGGYVYFEESAYSHQLSRADITTVSTF
IDLNITMLEDHEFVPLEVYTRHEIKDSGLLDYTEVQRRNQLHDLRFADIDTVIHADANAAMFAG
LGAFFEGMGDLGRAVGKVVMGIVGGVVSAVSGVSSFMSNP

Gingipain protease A (Porphyromonas gingivalis) – residues 228-720

YTPVEEKQNGRMIVIVAKKYEGDIKDFVDWKNQRGLRTEVKVAEDIASPVTANAIQQFVKQEYE
KEGNDLTYVLLVGDHKDIPAKITPGIKSDQVYGQIVGNDHYNEVFIGRFSCESKEDLKTQIDRT
IHYERNITTEDKWLGQALCIASAEGGPSADNGESDIQHENVIANLLTQYGYTKIIKCYDPGVTP
KNIIDAFNGGISLVNYTGHGSETAWGTSHFGTTHVKQLTNSNQLPFIFDVACVNGDFLFSMPCF
AEALMRAQKDGKPTGTVAIIASTINQSWASPMRGQDEMNEILCEKHPNNIKRTFGGVTMNGMFA
MVEKYKKDGEKMLDTWTVFGDPSLLVRTLVPTKMQVTAPAQINLTDASVNVSCDYNGAIATISA
NGKMFGSAVVENGTATINLTGLTNESTLTLTVVGYNKETVIKTINTNGEPNPYQPVSNLTATTQ
GQKVTLKWDAPSTKTNATTNTARSVDGIRELVLLSVSDAPELLRS

Outer surface protein A (Borrelia burgdorferi) – residues 17-273

CKQNVSSLDEKNSVSVDLPGEMNVLVSKEKNKDGKYDLIATVDKLELKGTSDKNNGSGVLEGVK
ADKSKVKLTISDDLGQTTLEVFKEDGKTLVSKKVTSKDKSSTEEKFNEKGEVSEKIITRADGTR
LEYTEIKSDGSGKAKEVLKSYVLEGTLTAEKTTLVVKEGTVTLSKNISKSGEVSVELNDTDSSA
ATKKTAAWNSGTSTLTITVNSKKTKDLVFTKENTITVQQYDSNGTKLEGSAVEITKLDEIKNAL
K

Outer surface protein C (Borrelia burgdorferi) – residues 11-191

GAMEASTNPDESAKGPNLTEISKKITDSNAVVLAVKEVEALLSSIDELAKTIGKKIEANGLGNE
ADKNGSLLAGAYAISTLIKQKLDGLKGLEGLNKEIAEAKKCSEAFTKKLQDSNADLGKHNATDA
DSKEAILKTNGTKTKGAKELEELFKSVESLSKAAKEALSNSVKELTSPVVAESPKKP

Global kinetic analysis of Aβ42 aggregation

The dominant aggregation process can be inferred reasonably well from the scaling of aggregation half-times with initial monomer concentration (i.e., $t_{0.5} \sim [M]_0^{\gamma}$, where γ is the scaling exponent (1). Interestingly in our case, the resulting double logarithmic plot was non-linear and displayed convex shape with the scaling exponent shifting from -1.6 at low monomer concentrations (0.5-3 μM) to -0.5 at high concentration regime (3-26 μM) (Figure 1c). The scaling coefficient of -1.6 is close to the -1.5 corresponding to secondary nucleation with nucleus size of n=2 (-(n+1)/2) described for Aβ42 under similar experimental conditions (2). The low monomer dependency of the aggregation (scaling factor -0.5) at the high concentration regime is consistent with monomer-independent fibril fragmentation or saturating secondary nucleation dominant mechanisms (Figure 1e).

Although fragmentation cannot be fully excluded, we argue that it is unlikely to significantly contribute to the overall aggregation rate since all experiments were done under quiescent conditions. Moreover, we did not observe any major differences in the fibril length distribution by AFM that would support major role of fragmentation (Supplementary Figure 4). In contrast, the saturation of secondary nucleation has been observed for Aβ40, A2V mutant variant of Aβ42, and for Aβ42 in medium ionic strength (3–5). To scrutinize between the two possible scenarios, we fitted the data globally to both models using online software tool AmyloFit (1). The model of multi-step (i.e., saturating) secondary nucleation could reproduce the data much more reliably than the one including fragmentation and secondary nucleation in parallel (**Chyba! Nenalezen zdroj odkazů.**d). To constrain a fitting space and obtain a good fit, the saturation concentration of monomer (K_m) was kept constant whilst the combined rate constants for primary ($k_n k_e$) and secondary nucleation ($k_2 k_e$) were allowed to vary. The best fits based on the mean squared error was obtained for K_m in the range of 1-2.5 μM which is lower than observed in the literature (> 6 μM (4)). Although some of the individual traces are not fitted accurately, the overall behavior of the kinetics is captured by the model remarkably well (Figure 1d). Based on this evidence we exclude fragmentation as a significantly contributing factor.

References

1. Meisl G, Kirkegaard JB, Arosio P, Michaels TCT, Vendruscolo M, Dobson CM, et al. Molecular mechanisms of protein aggregation from global fitting of kinetic models. Nat Protoc. 2016 Feb;11(2):252–72.

2. Cohen SIA, Linse S, Luheshi LM, Hellstrand E, White DA, Rajah L, et al. Proliferation of amyloid-β42 aggregates occurs through a secondary nucleation mechanism. Proc Natl Acad Sci USA. 2013 Jun 11;110(24):9758–63.

3. Meisl G, Yang X, Frohm B, Knowles TPJ, Linse S. Quantitative analysis of intrinsic and extrinsic factors in the aggregation mechanism of Alzheimer-associated Aβ-peptide. Sci Rep. 2016 Jan 13;6(1):18728.

4. Meisl G, Yang X, Dobson CM, Linse S, Knowles TPJ. Modulation of electrostatic interactions to reveal a reaction network unifying the aggregation behaviour of the Aβ42 peptide and its variants. Chem Sci. 2017;8(6):4352–62.

5. Meisl G, Yang X, Hellstrand E, Frohm B, Kirkegaard JB, Cohen SIA, et al. Differences in nucleation behavior underlie the contrasting aggregation kinetics of the Aβ40 and Aβ42 peptides. Proc Natl Acad Sci USA. 2014 Jul;111(26):9384–9.

Supplementary Tables

Supplementary Table 1: Antibodies and reaction conditions for immunohistochemistry

antibody	cat. number	manufacturer	dilution
DAPI	D9542	Merck	1:1000/blocking buffer
Anti-HSV-1 ICP4	Ab6514	Abcam	1:300/blocking buffer
Anti-HSV-1/2	ABIN283660	Antibodies Online	1:300/blocking buffer
β-Amyloid (D54D2)	8243S	Cell Signaling Technology	1:300/blocking buffer
Donkey Anti-Mouse AF488	A21202	Invitrogen	1:400/blocking buffer
Donkey Anti-Rabbit AF568	A10042	Invitrogen	1:400/blocking buffer
Goat Anti-Mouse AF647	A21235	Invitrogen	1:400/blocking buffer

Supplementary Figures

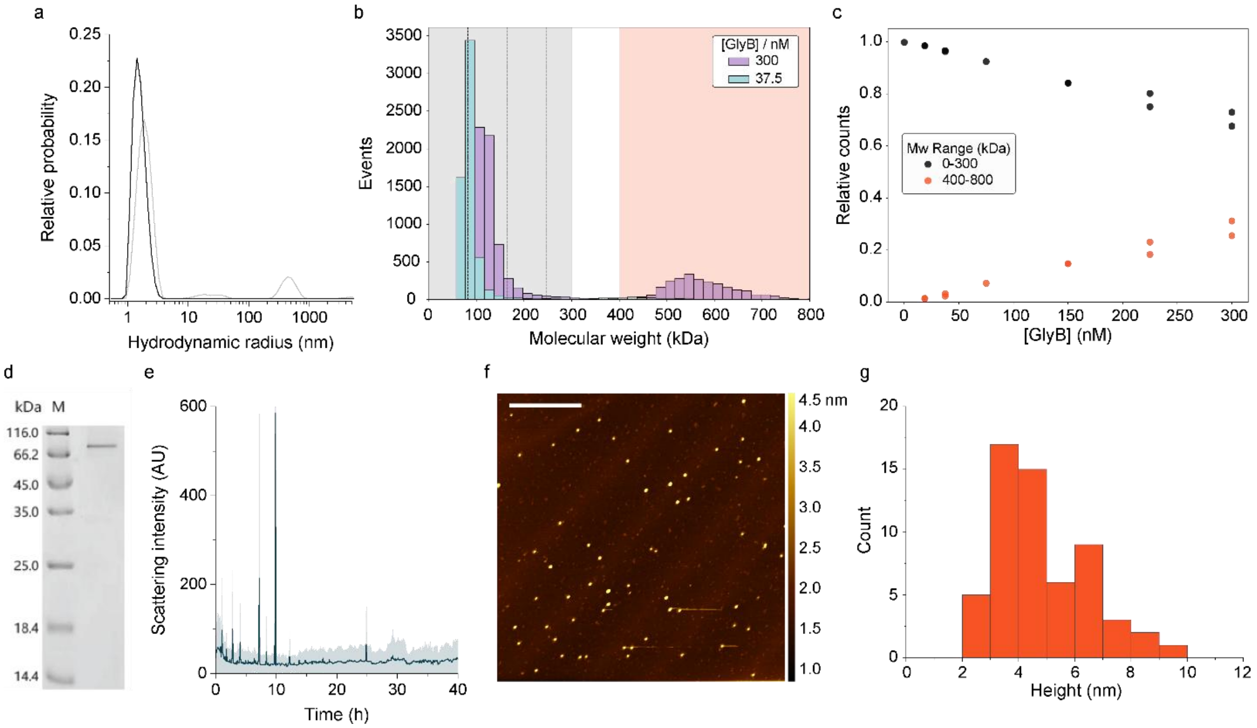

Supplementary Figure 1. Biophysical characterization of glyB. A) Dynamic light scattering of 2 µM glyB in 20 mM NaP 0.2 mM EDTA buffer pH 8 at 25 °C. The intensity- and mass-weighted size distributions derived from the fitting of the autocorrelation function are depicted by gray and black lines, respectively**. B) Size distribution of glyB oligomeric species** at 37.5 (cyan) and 300 nM (purple) protein concentration. The molecular weight range of the low and higher molecular weight species are shaded in black and orange, respectively. The vertical lines correspond to the theoretical Mw of monomer, dimer and trimer. **C) Concentration dependence of oligomeric species.** The points correspond to the relative counts from the two Mw ranges marked by shaded areas in (b) for each concentration studied. **D) SDS-PAGE analysis** of the glyB. **E) Static light scattering** of 2 µM glyB sample during 40h incubation at 37 °C. The blue line and the shaded area correspond to the mean ± SD from 2 independent measurements. **F) AFM analysis of the samples** retrieved from the capillary at the endpoint of the measurement showed in (e). The z-height is indicated by the color bar. Scale bar corresponds to 0.5 µm. **G) Distribution of particles heights** based on the AFM analysis shown in (f). The mean height of the particles (n=58) was determined to be 4.8 ±1.7 nm.

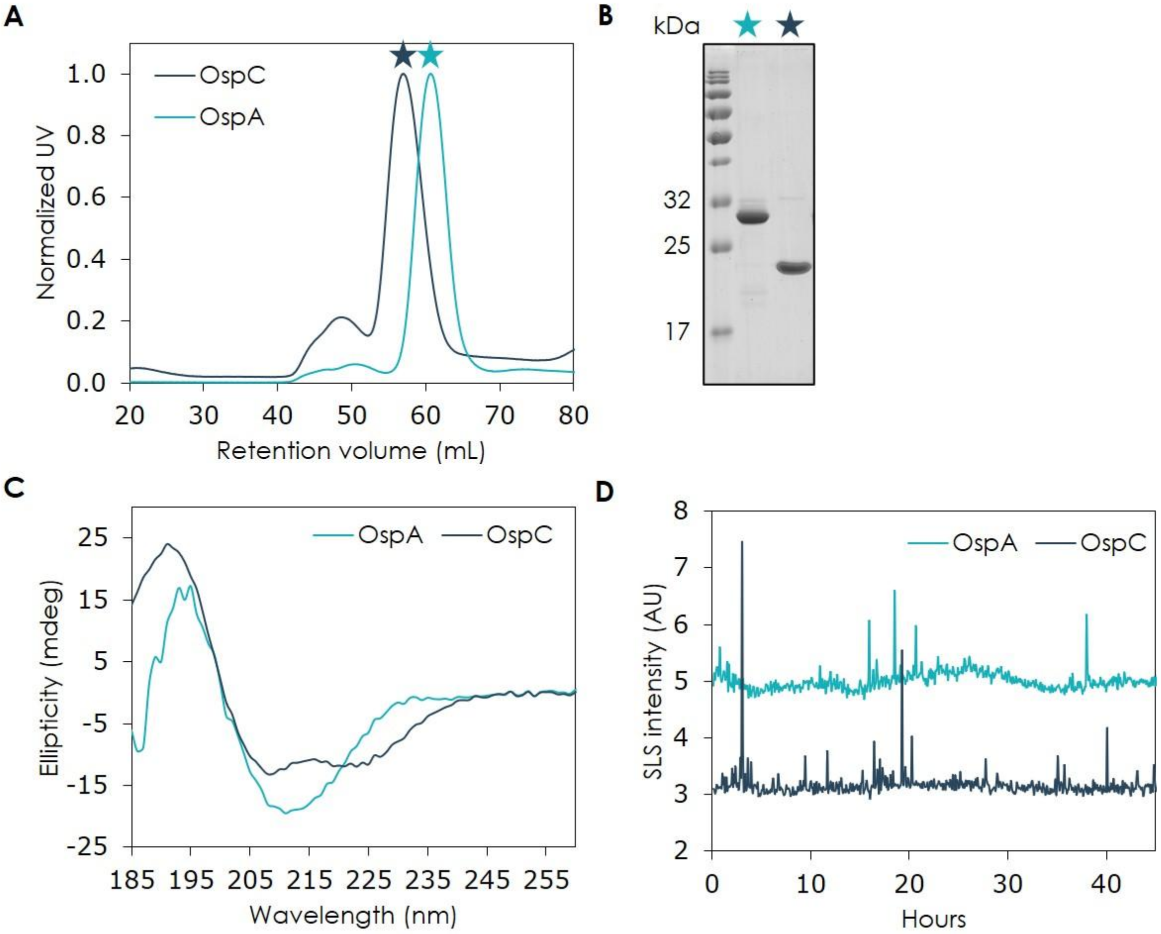

Supplementary Figure 2: Biophysical characterization of the OspA (cyan) and OspC (blue) produced recombinantly in-house. A) Size exclusion chromatography of the proteins after the His-tag cleavage. The peaks corresponding to the elution of target proteins are indicated by the stars. Only the central portion of the peak were used. B) SDS-PAGE analysis of the peak fractions from A. C) CD spectra of OspA and OspC confirming the predominantly beta-sheeted or alpha helical content of the two proteins, respectively. D) Colloidal stability of the OspA and OspC probed by the measurement of the static light scattering (SLS) at 266 nm at 37 °C. No aggregation of the proteins was observed over the concentration ranges and time courses used in this study. Representative curves at the highest measured concentration of both proteins are shown.

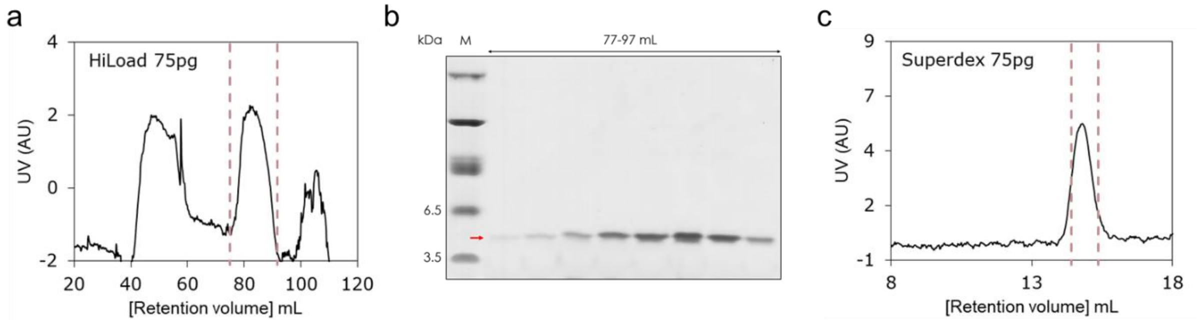

Supplementary Figure 3: Purification of recombinantly produced Aβ42. A) SEC analysis of the fraction containing Aβ42 after the anion-exchange. **B)** SDS-PAGE analysis of the SEC fractions indicated by the dashed lines in A. **C)** Isolation of the monomeric Aβ42 prior to the experiments. Fractions containing the highest amount of Aβ42 after the SEC (shown in B) were lyophilized for storage. Before the experiment, lyophilized powder was dissolved in 6M GndHCl and monomeric Aβ42 isolated by SEC using Superdex 75pg column. The central portion of the peak (marked with dashed lines) was collected into a pre-chilled low-binding tube on ice and used immediately for the experiments.

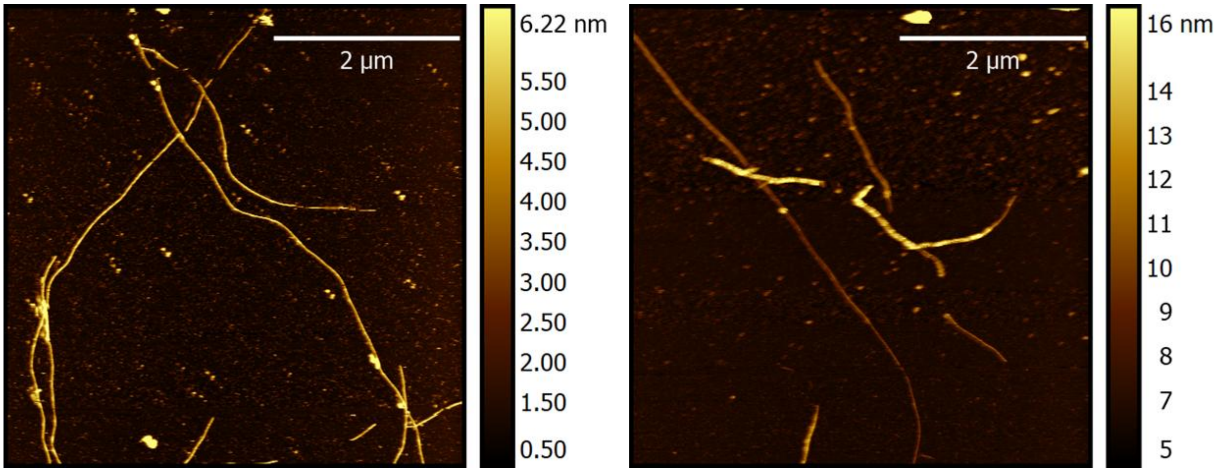

Supplementary Figure 4 AFM images of Aβ42 fibrils. The images are color-coded according to the z-height scale shown on the right side of the pictures.

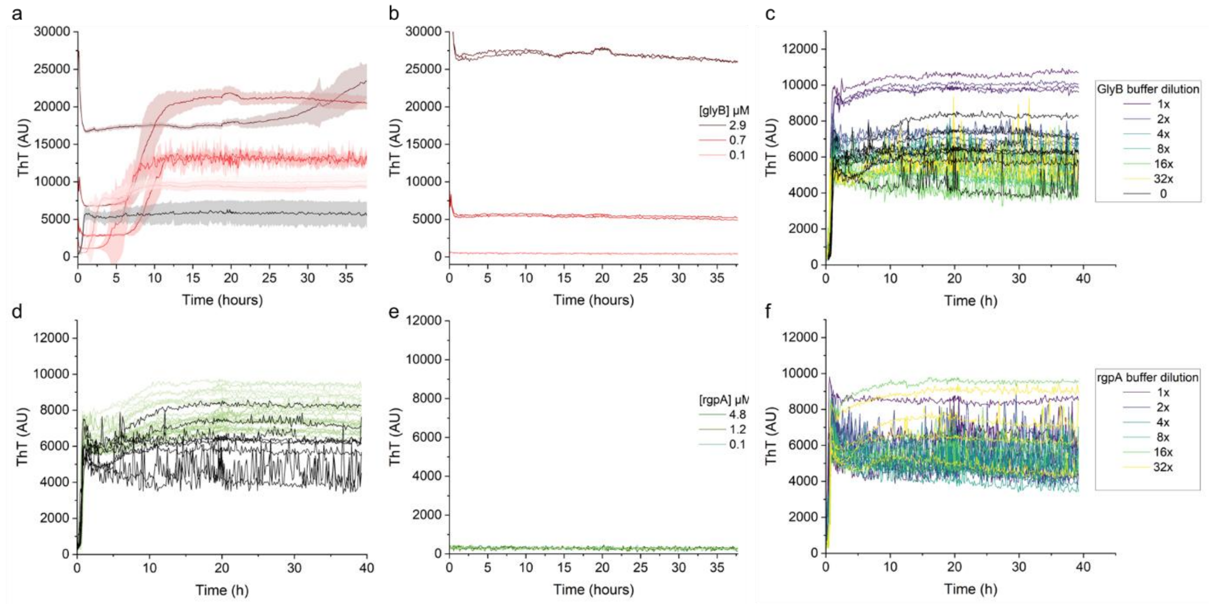

Supplementary Figure 5 Raw and control experiments for glyB (top) and rgpA (bottom). A) and D) Depict raw ThT data of glyB and rgpA, respectively, in the presence of Aβ42 corresponding to those in **Chyba! Nenalezen zdroj o dkazů.**d. **B) and E)** show three different concentrations of glyB and rgpA, respectively, in the absence of Aβ42. The background fluorescence of glyB was subtracted from traces in a during the analysis. **C) and F)** Effect of the storage buffer to aggregation of Aβ42. The commercial samples of rgpA and glyB were supplied in respective storage buffers. Their effect to Aβ42 was verified by preparing their dilutions in the absence of the proteins and presence of the Aβ42 at the same concentration used in the other experiments (i.e., 7.2 μM).

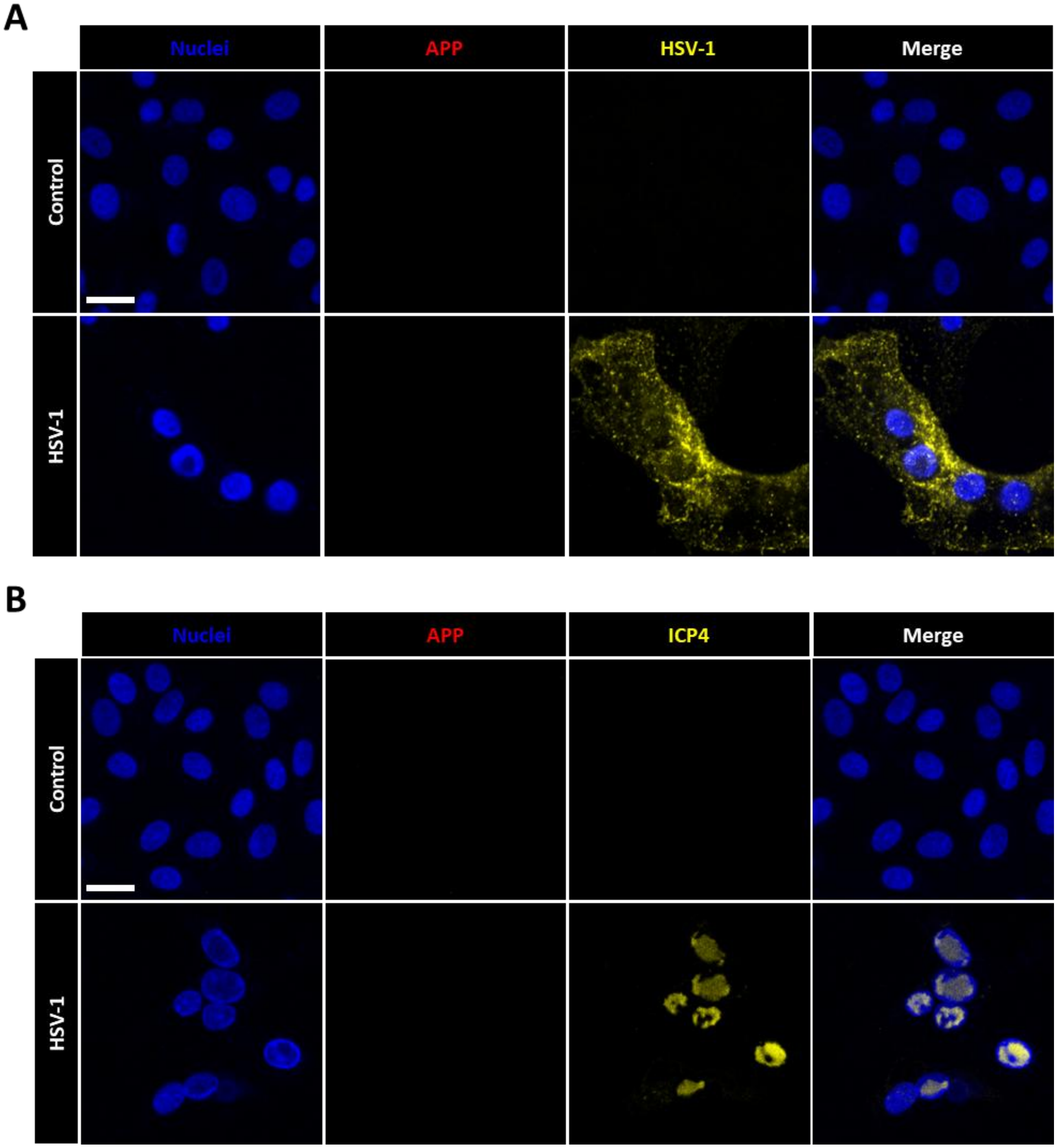

Supplementary Figure 6. Verification of β-Amyloid Antibody Specificity Against HSV-1. Vero cells (African green monkey kidney) were infected with HSV-1 (MacIntyre strain) at a multiplicity of infection (MOI) of 1 for 24 hours. Viral infection was confirmed using specific antibodies against (A) HSV-1/2 (yellow) and (B) ICP4 (yellow). No non-specific binding of the β-Amyloid antibody (clone D54D2, red) to HSV-1-infected cells was observed. Non-infected cells were used as control. Immunofluorescence was performed using the following marker combinations: β-Amyloid (red) with either ICP4 (yellow) or HSV-1 (yellow), and DAPI (blue) for nuclear staining. Scale bar: 20 μm.

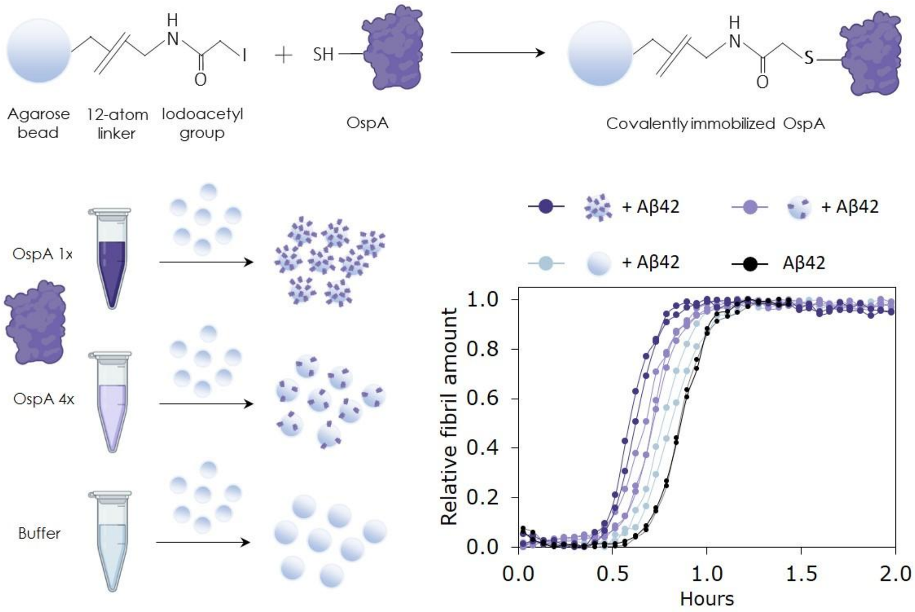

Supplementary Figure 7: Immobilization of OspA and its effect on Aβ42. OspA was covalently immobilized to agarose beads by coupling its N-terminal cysteine with iodoacetyl group. The unreacted groups and beads in the negative control (buffer) were blocked by coupling with L-cysteine. Different initial concentrations of OspA were used for the immobilization to generate beads with varying number of immobilized protein molecules on the surface. Final concentrations of the immobilized OspA were 17.2, 8.6 and 4.3 µM. Lag time of aggregation of Aβ42 (black) was increased in the presence of the beads (blue) and even more with increasing concentration of immobilized OspA (purple).
